# Pyruvate dehydrogenase autoantibodies in autoantibody‐negative patients with seizures are associated with reduced pyruvate dehydrogenase activity

**DOI:** 10.1002/epi.70282

**Published:** 2026-05-14

**Authors:** Annika Breuer, Chiara A. Hummel, Tobias Baumgartner, Juliane L. Berns, Afaf Milad Said, Isabel Bünger, Susanne Schoch, Gábor Zsurka, Harald Prüss, Wolfram S. Kunz, Rainer Surges, Albert J. Becker, Julika Pitsch

**Affiliations:** ^1^ Department of Epileptology University Hospital Bonn, University of Bonn Bonn Germany; ^2^ Institute of Experimental Epileptology and Cognition Research University Hospital Bonn, University of Bonn Bonn Germany; ^3^ Department of Pediatric Neurology Charité Universitätsmedizin Berlin Berlin Germany; ^4^ Department of Neurology and Experimental Neurology Charité Universitätsmedizin Berlin Berlin Germany; ^5^ Institute of Cellular Neurosciences II, University Hospital Bonn University of Bonn Bonn Germany; ^6^ German Center for Neurodegenerative Diseases Berlin Berlin Germany

**Keywords:** antimitochondrial autoantibodies, enzyme activity, mitochondria, psychiatric symptoms, seizures

## Abstract

**Objective:**

We investigated the presence and potential functional relevance of antimitochondrial autoantibodies in patients suspicious for autoimmune encephalitis (AIE) associated with psychiatric symptoms and/or seizures, who were negative for known antineuronal autoantibodies.

**Methods:**

We screened serum samples from 387 patients autoantibody‐negative for known antineuronal autoantibodies with psychiatric disturbances and/or epileptic seizures, including patients with temporal lobe epilepsy of unknown etiology. Various techniques, including immunoblotting, immunoprecipitation, mass spectrometry, immunohistochemistry, and in vitro assays assessing neuronal autoantibody uptake, neuronal viability, pyruvate dehydrogenase (PDH) enzyme activity, and mitochondrial DNA levels in biofluids were applied.

**Results:**

Mass spectrometry detected all three subunits of the intramitochondrial PDHc—pyruvate dehydrogenase, dihydrolipoyl acetyltransferase, and dihydrolipoyl dehydrogenase—as targets of antibodies present in serum samples from three index patients suspicious for AIE with psychiatric symptoms or seizures. The presence of the anti‐PDHc autoantibodies was confirmed by immunoblotting in 12 of 387 patients. Exposure of cultured primary neurons to commercial anti‐PDH antibodies resulted in neuronal uptake and loss of neuronal viability. Patient‐derived autoantibodies also impaired PDH enzyme activity in vitro. Additionally, cell‐free mitochondrial DNA fragment levels were elevated in the serum and cerebrospinal fluid of PDH‐positive patients compared to controls.

**Significance:**

Anti‐PDH autoantibodies were detected in patients suspicious for AIE with seizures and/or psychiatric symptoms as core manifestation in the absence of known antineuronal autoantibodies. These autoantibodies bind neuronal structures and reduce PDH enzyme activity under experimental conditions, supporting mechanistic plausibility of a functional role in disease.

**Key points**

- Anti‐PDH autoantibodies are detected in patients presenting with seizures and/or psychiatric symptoms.
- Patient‐derived anti‐PDH autoantibodies bind to excitatory and inhibitory neurons in murine tissue.
- Anti‐PDH autoantibodies reduce PDH enzyme activity under permeabilized in vitro conditions.
- Cell‐free mitochondrial DNA fragments are elevated in serum of anti‐PDH‐positive patients.

## 1 INTRODUCTION

Mitochondrial dysfunction is increasingly recognized to be linked to epilepsy, as both clinical and experimental evidence suggest a vicious circle between impaired energy metabolism and seizures. Clinically, syndromes such as MELAS (mitochondrial encephalopathy, lactic acidosis, and strokelike episodes) and MERRF (myoclonic epilepsy with ragged red fibers) underline that those mitochondrial defects can directly contribute to the development of epilepsy.^1^ Moreover, seizures are reported in 35%–60% of patients with confirmed mitochondrial disease,^2^ and mitochondrial dysfunction is noted in many patients with refractory epilepsy.^3^ Experimentally, seizures have been shown to impair mitochondrial function, leading to neuronal death and promoting epileptogenesis.^4^, ^5^, ^6^ Additionally, electrophysiological data show that impaired energy metabolism increases brain excitability and abnormal neuronal firing.^7^

Several mechanisms link mitochondrial dysfunction to seizures, such as disruptions in calcium homeostasis, oxidative stress, and reduced network inhibition, especially in mitochondria‐rich interneurons.^8^ Fast‐firing neurons, such as parvalbumin‐positive interneurons, are particularly vulnerable to mitochondrial dysfunction due to their high energy demands, and loss of interneurons has been shown to induce seizures.^9^

Despite these findings, the underlying mechanisms leading to seizures in mitochondrial diseases remain poorly understood.^3^ There are few systematic studies examining the link between epilepsy and mitochondrial disease,^10^, ^11^ and none consider autoimmune processes.

The pyruvate dehydrogenase complex (PDHc) is located in the mitochondrial matrix and essential for mitochondrial energy production in neurons. Therefore, PDHc deficiency, which disrupts adenosine triphosphate (ATP) production and leads to elevated lactic acid, is associated with severe neurological symptoms, including epilepsy.^12^ The severity of symptoms correlates with the level of PDHc activity, with lower activity causing earlier and more extensive brain damage. PDHc deficiency may also affect neurotransmitter release through disruptions in calcium regulation.^13^ Thus, PDHc deficiency may play a critical role in linking mitochondrial disease and epilepsy. However, the detailed pathogenic role of PDHc in epilepsy syndromes is still enigmatic.

Here, we report on a novel association of anti‐PDH autoantibodies (Abs) in patients suspicious for autoimmune encephalitis (AIE) presenting with psychiatric symptoms and/or seizures. Using a combination of in vitro analyses, we explore the potential functional relevance of anti‐PDH Abs in this clinical context.

## 2 MATERIAL AND METHODS

### 2.1 Human samples

Sera samples of 387 patients who presented to the Department of Epileptology, University Hospital Bonn, Germany, between 2017 and 2021 were included in this study. Inclusion criteria were (1) clinical suspicion of AIE, manifested either with psychiatric symptoms and/or epileptic seizures including patients with temporal lobe epilepsy (TLE) of unknown etiology^14^; (2) absence of established antineuronal Abs previously associated with AIE; and (3) presence of at least one additional band in the routine Western blot (WB) screenings with a size smaller than 110 kDa, indicating the presence of novel Abs.^15^ The control group comprised (1) sera of 31 healthy donors (male: *n* = 14, female: *n* = 17; mean age = 33.6 ± 10.2 SD) showing no reactivity in immunoblot screening, (2) sera of 100 so far Ab‐negative for antineuronal Abs patients suspected for AIE without an indication of unknown antibodies on WB or brain slides, and (3) sera from patients positive for anti‐glutamic acid decarboxylase 65‐kD isoform (GAD65; *n* = 6) and anti‐leucine‐rich glioma‐inactivated 1 (LGI1; *n* = 6). Intrathecal synthesis of antimitochondrial antibodies (AMAs) was not systematically assessed, as antibody‐specific indices were not available for all patients.

### 2.2 Ab screening

All serum and cerebrospinal fluid (CSF) samples were systematically screened for established neuronal surface and onconeuronal Abs associated with AIE and paraneoplastic neurological syndromes. Tested antibodies included amphiphysin, CV2 (collapsing response mediator protein 5; CRMP5), PNMA2 (paraneoplastic Ma2; Ma2/Ta), Ri (antineuronal nuclear antibody type 1; ANNA‐2), Yo (Purkinje cell cytoplasmic antibody type 1; PCA‐1), Hu (antineuronal nuclear antibody type 1; ANNA‐1), rRecoverin, SOX1 (SRY‐box transcription factor 1), Titin, Zic4 (zinc finger protein of the cerebellum 4), GAD65 (glutamic acid decarboxylase 65 kDa isoform), and Tr (DNER; delta/notch‐like EGF repeat‐containing receptor), as well as surface‐directed antibodies against NMDAR (N‐methyl‐D‐aspartate receptor), CASPR2 (contactin‐associated protein‐like 2), LGI1 (leucine‐rich glioma‐inactivated 1), GABAAR (gamma‐aminobutyric acid type A receptor), GABABR (γ‐aminobutyric acid type B receptor), AMPAR (α‐amino‐3‐hydroxy‐5‐methyl‐4‐isoxazolepropionic acid receptor), and GAD65. Detailed assay methodology, dilutions, and manufacturer specifications are provided in the Data S1.

Immunoblotting on brain lysates was used to screen for novel Abs in serum and CSF of Ab‐negative patients. Protein lysates of rat and mouse brain tissue, surgical human hippocampal tissue of patients suffering from pharmacoresistant TLE, and murine crude synaptosomes were isolated as described previously.^16^

### 2.3 Immunoprecipitation and mass spectrometry

Immunoprecipitation was performed as recently described^16^ using whole mouse brain tissue with two replicates of every biosample. Samples were run in parallel on two applications of sodium dodecyl sulfate–polyacrylamide gel electrophoresis (SDS‐PAGE), one of which was stained with Roti‐Blue 5× (Roth, A152.1); the other one was used for immunoblotting. After membrane staining, additional bands detected in immunoblot not present in controls were cut out from gels of corresponding replicates and analyzed via mass spectrometry by comparing their abundance ratios to controls.^17^

### 2.4 Validation of anti‐PDHc Ab binding in WB

PDH from porcine heart (Sigma‐Aldrich, P7032‐10 U; .625 mg/mL in ddH_2_O), was denatured while heating with 6× Laemmli buffer for 10 min at 95°C and loaded for SDS‐PAGE. Gels were blotted on nitrocellulose membranes, blocked, and washed as described above. Proteins were incubated with sera (1:100), CSF (1:1), or commercial murine monoclonal anti‐PDHc antibody cocktail followed by appropriate secondary antibody (Table S1). Titer of anti‐PDHc Abs in biosamples was analyzed by using ascending titration series (1:100 to 1:100 000).

### 2.5 Immunohistochemistry on mouse brain slices

Adult C57Bl/6N (Charles River) mice were sacrificed under isoflurane anesthesia. Brains were removed, embedded in paraformaldehyde (PFA), sectioned (4 μm), and dried overnight at 37°C and processed as described before.^18^ After washing and blocking, slices were incubated with commercial murine monoclonal anti‐PDHc antibody cocktail and either patients' anti‐PDH Ab‐positive serum, serum of healthy controls, or normal human serum (1:100) overnight at 4°C. The following day, slices were incubated in secondary antibody (Table S1) and 4,6‐diamidino‐2‐phenylindole (Sigma‐Aldrich D9542, 1:200) for 2 h at room temperature (RT).

### 2.6 Primary hippocampal neurons

Dissociated primary hippocampal neurons (PHNs) of embryonic day (E) 15–19 murine hippocampi (C57Bl6/N) were prepared as described previously and cultured in Neurobasal medium.^16^, ^19^ A subset of neurons was virally transduced on day in vitro (DIV) 5 with either mDlx‐jGCAMP6f (rAAV2/1‐mDLX‐jGCAMP6f‐Fishell‐2, Addgene #83899) or vGLUT2‐mCherry (rAAV2/1‐VGlut2‐mCherry [vesicular‐glutamate transporter 2; expressed in excitatory neurons; plasmid kindly provided by M. P. Anderson, Harvard University]; recombinant adeno‐associated virus [rAAVs] purchased from Virus Core Facility of the Medical Faculty of Bonn University) to visualize inhibitory and excitatory neurons, respectively. For each biological culture replicate, one litter of mouse embryos was used. From each litter, several experiments including PHNs were conducted.

### 2.7 Immunocytochemistry on murine PHNs

On DIV14, PHNs were fixed in 4% PFA for 10 min and washed with phosphate‐buffered saline (PBS) three times. Neurons were permeabilized with .3% triton for 10 min and blocked with 1% bovine serum albumin (BSA), 10% normal goat serum (NGS), and .2% triton for 1 h at RT before incubating with appropriate primary Abs at 4°C overnight. Cells were washed with PBS three times prior to incubation with secondary Abs (Table S1) for 1 h at RT followed by three washing steps, mounting (Mowiol), and storage at 4°C. Neuronal staining was performed in three biological replicates consisting of four technical replicates each.

### 2.8 Anti‐PDH antibody uptake and neuronal viability assays

On DIV12–14, PHNs were incubated with 20 μg/mL commercial PDH antibodies (cPDH) for 20 min, in a time series of 4 h from 4 h to 24 h, or 48 h and fixed in 4% PFA for 10 min after three washing steps with PBS. Commercial anti‐PDH Abs were used as standardized reagents to assess antibody‐mediated effects under controlled conditions, as sufficient patient‐derived IgG material was not available for all functional assays. The antibody concentration was selected based on commonly used conditions in in vitro neuronal antibody assays and approximates total physiological CSF IgG concentrations (20–40 μg/mL), while accounting for the absence of dynamic exposure conditions and immune cofactors in vitro.^20^, ^21^ Neurons were permeabilized with .3% triton for 10 min and blocked with 1% BSA, 10% NGS, and .1% triton for 10 min at RT. To visualize postsynaptic locations, neurons were incubated with anti‐homer1 in blocking solution overnight at 4°C. Cells were washed three times with PBS prior to incubation with secondary antibody (Table S1) for 1 h at RT followed by three PBS washing steps, mounting (Mowiol), and storage at 4°C. For neuronal viability analysis, PHNs were transduced on DIV4 with hSyn‐GFP (rAAV2/1‐hSyn‐GFP) prior to Ab incubation as described above. Neuronal uptake and viability were determined in four biological replicates consisting of three technical replicates each.

### 2.9 Enzyme assay

Mitochondria were isolated from HEK 293 cells as described before,^22^, ^23^ with small modifications of the composition of isolation buffer (IB) + BSA (210 mmol·L^−1^ mannitol, 70 mmol·L^−1^ sucrose, 5 mmol·L^−1^ hydroxyethylpiperazine ethane sulfonic acid KOH + .25% BSA) and the final centrifugation steps (30 min at 10 000 × *g* and 15 min at 10 000 rpm). The final pellet was resuspended in 900 μL cold IB + protease inhibitor (Roche, 5892791001). The activity of PDH was measured according to the protocol of the PDH Activity Assay Kit (Sigma‐Aldrich, MAK183‐1KT). For all samples, 5 μL of isolated mitochondria was used. The concentration of commercial Abs was 10 μg/μL, whereas patient CSF was used at a dilution of 1:2. Intratect and no treatment were used as controls in all experiments in addition to the positive control provided in the kit. All samples were plated onto a 96‐well flat bottom plate and measured with the Tecan Infinite 200 Pro plate reader. Measurements were taken from five biological replicates of isolated mitochondria consisting of three technical replicates for commercial Abs and from three biological replicates with three technical replicates for CSF experiments. The assay employs proprietary buffers containing mild detergents that permeabilize mitochondrial membranes to allow substrate and reagent access to the matrix‐localized PDHc. Accordingly, mitochondrial outer and inner membrane integrity is intentionally disrupted during the assay procedure. Therefore, this assay measures maximal enzymatic activity under permeabilized conditions and does not reflect physiological antigen accessibility within intact mitochondria. The system is designed to assess intrinsic PDH catalytic function rather than antigen accessibility within intact mitochondria.

The assay conditions do not model in vivo mitochondrial membrane integrity and should not be interpreted as reflecting physiological antibody access to matrix‐localized PDH in intact cells.

### 2.10 Quantitative polymerase chain reaction

DNA was isolated with the QiAamp DNA Mini and Blood Mini Kit (QIAGEN) following the Body Fluid Spin Protocol for low‐concentration samples. Quantitative polymerase chain reactions (qPCR) were performed on the CFX qPCR system (Bio‐Rad) using iTaq Universal Probes Supermix (Bio‐Rad) with the following primers: MT16520_F (5′‐CATAAAGCCTAAATAGCCCACACG‐3′) and MT35_R (5′‐TCCCGTGAGTGGTTAATAGGGTGAT‐3′) with TaqMan probe MT_TM 5′‐FAM‐AGACATCACGATGGATCACAGGTCT‐TAM‐3′ for mitochondrial DNA (mtDNA) and KCNJ10_F (5′‐GCGCAAAAGCCTCCTCATT‐3′) and KCNJ10_R (5′‐CCTTCCTTGGTTTGGTGGG‐3′) with TaqMan probe KCNJ10_TM (5′‐FAM‐TGCCAGGTGACAGGAAAACTGCTTCAG‐TAM‐3′). Amplification conditions were as follows: 95°C for 15 min, and 55 cycles of 95°C for 15 s and 62.5°C for 30 s. C_T_ values were defined at the inflection points of fitted sigmoid curves (four‐parameter Chapman curves). Absolute mtDNA copy numbers were calculated by comparing C_T_ values for mtDNA to a calibration curve obtained by determining cycle threshold (C_T_) values for the single‐copy nuclear reference gene *KCNJ10* in serial dilutions of genomic DNA.

### 2.11 Images

All images were captured using a laser‐scanning Nikon A1/T1 confocal microscope with a Plan APO IR 60× WI objective (numerical aperture = 1.27). Data were processed using Nikon NIS‐Elements 4.0 acquisition software.

### 2.12 Statistics

Experiments were conducted in a randomized and blinded manner. Statistical analyses were completed with GraphPad Prism 10.0.02 software. Sample size (*n*) for each experiment was calculated using power analysis, with parameters set within the accuracy of the individual experiment. All data are plotted as mean ± SD. Kruskal–Wallis and one‐way analysis of variance (ANOVA) with Dunn correction and two‐way ANOVA with Tukey correction were performed. Values were considered significant at *p* < .05.

## 3 RESULTS

### 3.1 Abs targeting the PDHc in patients suspected for AIE

**FIGURE 1:**
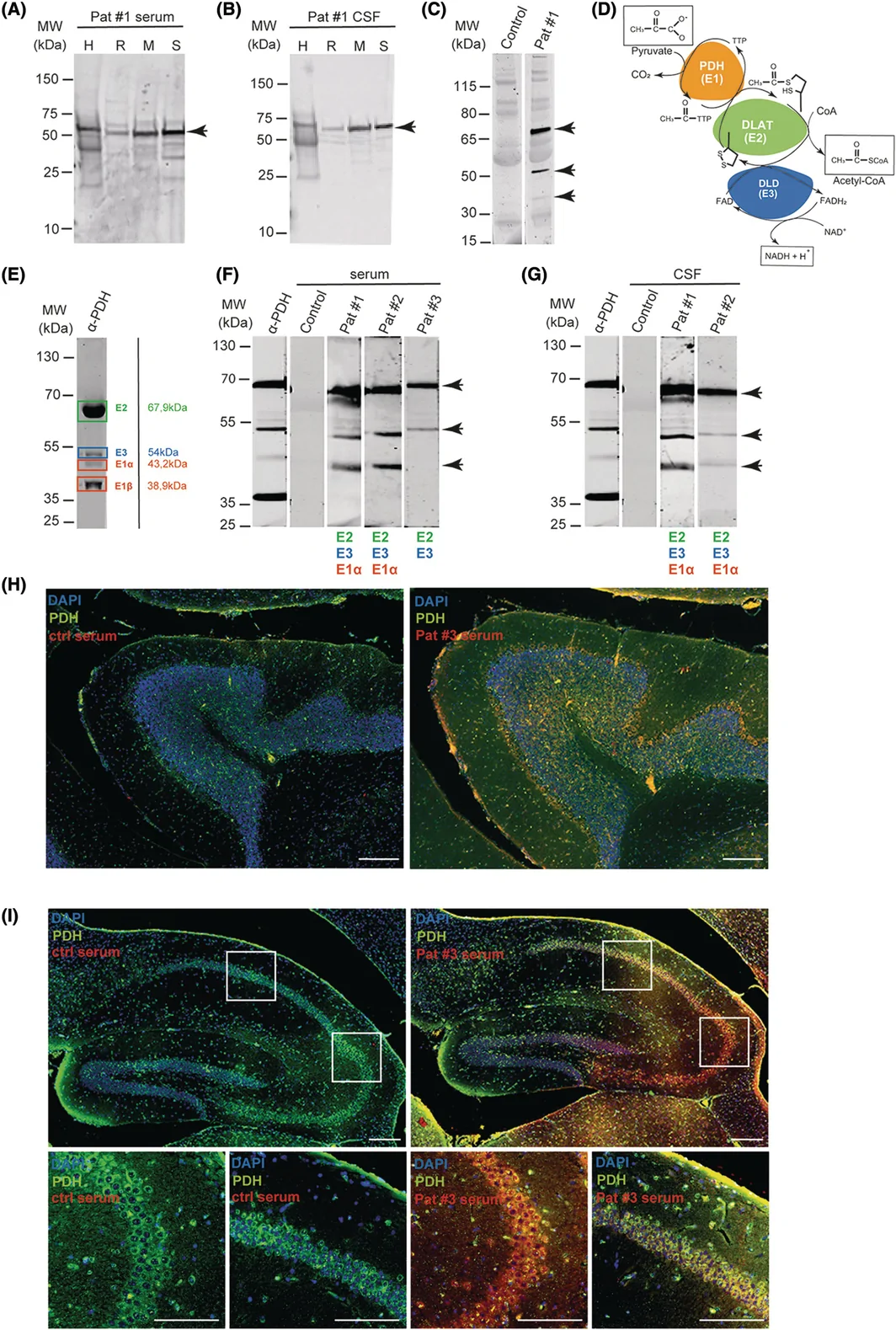
Pyruvate dehydrogenase complex (PDHc) subunits were identified as targets of autoantibodies derived from patients autoantibody‐negative for known autoimmune encephalitis autoantibodies. (A) Immunoblots of an index patient's serum (Patient [Pat] 1) and (B) cerebrospinal fluid (CSF) incubated with brain lysate of human (H), rat (R), mouse (M), and mouse synaptosomes (S) revealed a readily discernible binding pattern (arrow). (C) Immunoblot after immunoprecipitation performed with patient serum (Pat 1) represents additional signals (arrows) absent in the screening negative healthy control, identified by mass spectrometry as different subunits of the PDHc. (D) Schematic composition of PDHc showing interactions of its E1, E2, and E3 subunits and its corresponding chemical reactions of pyruvate decarboxylation that converts pyruvate into acetyl‐CoA. It represents the key step in the initiation of the citric acid cycle. (E) Immunoblot of PDHc from porcine heart showed the size of different subunits (E2, 67.9 kDa; E3, 54 kDa; E1α, 43.2 kDa; E1β, 38.9 kDa) detected by commercial antibodies against subunit proteins. (F) Sera of the three index patients showed reactivity against E2, E3, E1α (Pat 1, Pat 2) or rather E2 and E3 (Pat 3; arrows) of purified porcine heart PDHc protein. (G) CSF of Pat 1 and Pat 2 showed reactivity against the same subunits identified by corresponding sera (arrows). Representative images of index patients' serum incubated on mouse brain slices are shown. (H) Sera of Pat 3 and mouse monoclonal anti‐PDHc antibody cocktail both showed strong reactivity within granular cells of the cerebellum. In contrast, no binding pattern was detectable on mouse brain slices with sera of autoantibody‐negative human control. (I) Sera of Pat 3 and mouse monoclonal anti‐PDHc antibody cocktail both showed strong labeling within the hippocampal formation, especially in the CA1 and CA3 regions. No staining was visible in the negative control, using autoantibody‐negative human serum. Scale bars: 200 μm. ctrl, control; DAPI, 4,6‐diamidino‐2‐phenylindole; DLAT, dihydrolipoyl transacetylase; DLD, dihydrolipoamide dehydrogenase; FAD, Flavin adenine dinucleotide; NAD, nicotinamide adenine dinucleotide; TPP, Thiamine Pyrophosphate; MW, molecular weight.

Index Patient 1 presented with focal to bilateral tonic–clonic seizures at admission to our department. The diagnostic workup revealed elevated CSF protein levels, left temporal sharp waves on electroencephalogram, and a T2/fluid‐attenuated inversion recovery hyperintensity of the left amygdala on cranial magnetic resonance imaging. These findings were suggestive for autoimmune limbic encephalitis. Although this patient tested Ab‐negative for known antineuronal Abs associated with AIE, both serum and CSF samples were positive in immunoblot screening, showing synaptic neuronal binding on multiple brain lysates. This indicated the presence of brain protein‐specific Abs (Figure 1A,B). To identify the ligand of the Abs, we performed immunoprecipitation (Figure 1C) and mass spectrometry using 55 patient samples with similar findings in screening assays, which identified the PDHc as an antigen of Abs in three index patients (Table S2). Next, an immunoblot screening was established to identify all four subunits of the PDHc (E2, E3, E1α, and E1β; Figure 1D,E). These specific Abs were subsequently confirmed in the serum and available CSF samples of the index patients (Figure 1F,G).

### 3.2 Identification of the PDHc as Ab target in patients with psychiatric symptoms or seizures

Following the identification of Abs targeting the PDHc, we screened a cohort of 387 patients with a differential diagnosis of AIE, all presenting with psychiatric symptoms and/or seizures. Twelve patients (see Table S3 for clinical data) were seropositive for distinct subunits of the PDHc, as determined by immunoblot analysis using purified PDHc (positive for E2, E1α and/or E3). Abs titres in serum ranged from 1:100 to 1:250 000. Of the seven patients with available CSF, two were positive for anti‐PDH Abs.

### 3.3 Patient‐derived anti‐PDH Abs target both excitatory and inhibitory neurons

**FIGURE 2:**
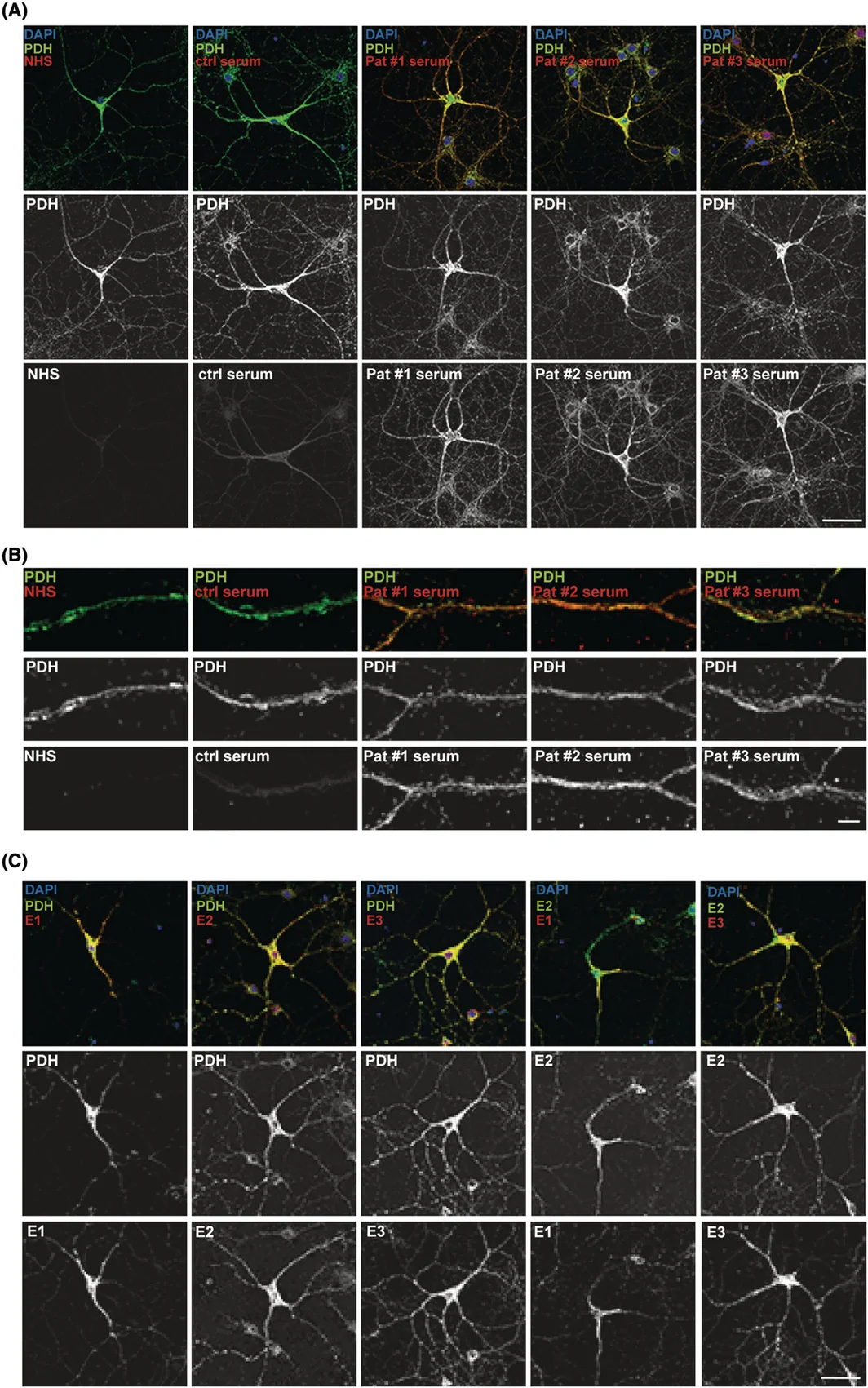
Anti‐pyruvate dehydrogenase complex (PDHc) autoantibody‐positive patients' sera strongly labeled murine primary hippocampal neurons and colocalized with commercial anti‐PDHc antibody. Representative images show serum of index Patients [Pat] 1–3 incubated on primary hippocampal neurons. (A) Sera of Patients 1–3 showed strong colocalization with commercial mouse monoclonal anti‐PDHc antibody cocktail on primary hippocampal neurons in the soma and on dendrites. In contrast, no colocalization was detectable using serum of autoantibody‐negative healthy control or commercial normal human serum (NHS; scale bar: 50 μm). (B) Anti‐PDHc autoantibody‐positive patients' sera and mouse monoclonal anti‐PDHc antibody cocktail strongly labeled dendrites, in which PDH is enriched in mitochondria (scale bar: 5 μm). (C) Mouse and rabbit monoclonal antibodies against different subunits of PDHc (E1a, E2, E3) showed strong colocalization among each other, indicating local affiliation of all three proteins to one complex (scale bar: 50 μm). ctrl, control; DAPI, 4,6‐diamidino‐2‐phenylindole.

Immunohistochemistry on mouse brain sections confirmed the presence of anti‐PDH Abs in patient sera. Sera from PDH antibody‐positive patients showed intense reactivity in the cerebellar Purkinje cell layer (Figure 1H) and strong binding in the hippocampal CA1 and CA3 pyramidal cell layer (Figure 1I), corresponding to the widespread distribution of PDH in these brain areas. To determine a neuronal binding pattern of anti‐PDH Abs, PHNs were stained with patient sera. The sera of index patients displayed strong localization of PDH in the somata (Figure 2A) and neural arbors (Figure 2B). Commercial antibodies targeting various PDHc subunits colocalized with PDH in both soma and dendrites, indicating ubiquitous expression of the complex in neuronal tissue (Figure 2C).

**FIGURE 3:**
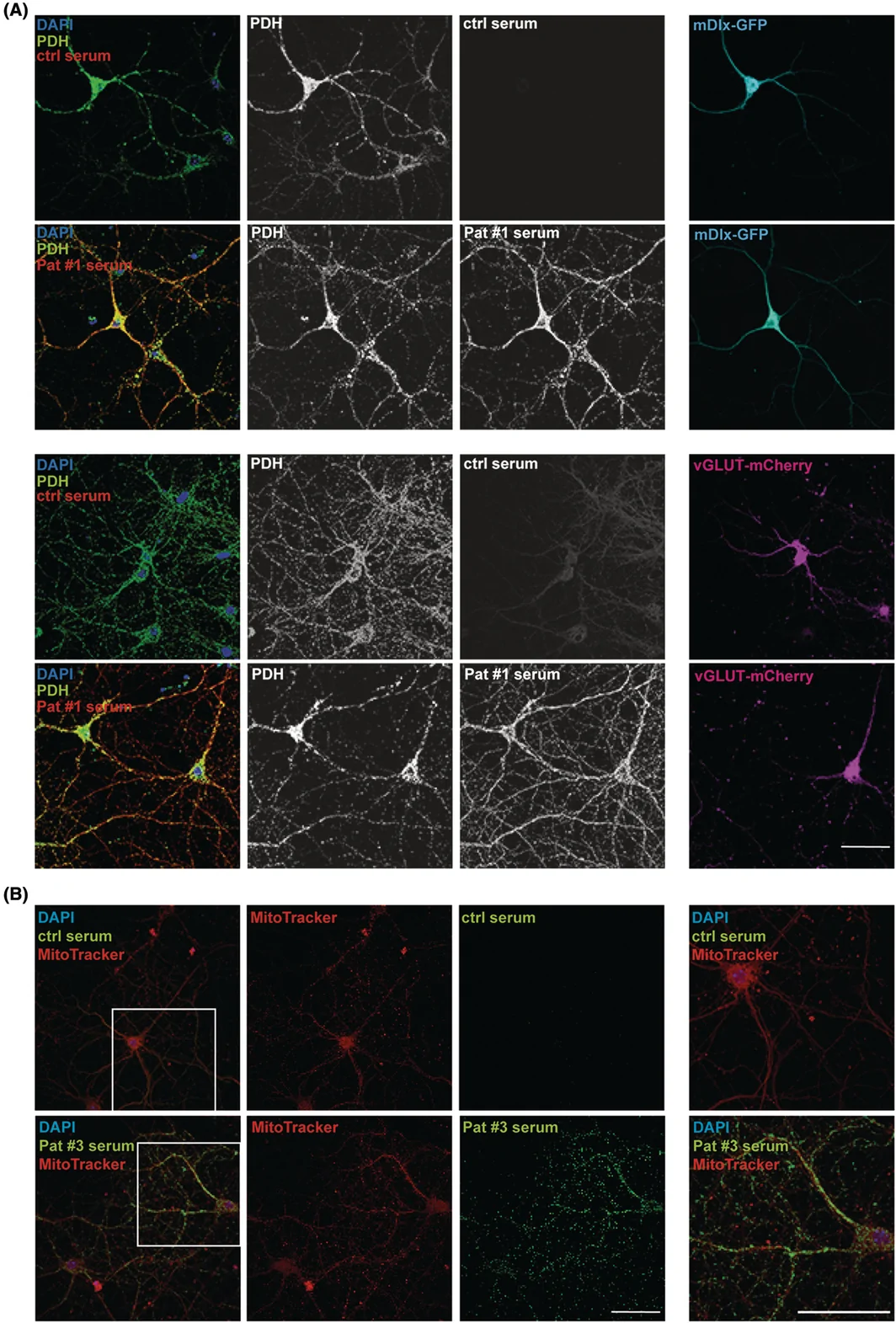
Anti‐pyruvate dehydrogenase complex (PDHc) autoantibody‐positive sera showed strong reactivity with inhibitory and excitatory neurons, localized to mitochondria. (A) Representative images of Patient (Pat) 1 serum incubated on transduced primary hippocampal neurons (PHNs). Anti‐PDHc autoantibody‐positive serum showed strong colocalization with commercial mouse monoclonal anti‐PDHc antibody cocktail on mDlx‐GFP‐transduced primary hippocampal neurons, indicating presence of PDHc in inhibitory interneurons. Autoantibody‐negative human serum showed no colocalization with mDlx‐GFP‐transduced neurons. Anti‐PDHc autoantibody (Ab)‐positive serum also showed strong colocalization with commercial mouse monoclonal anti‐PDHc antibody cocktail on vGLUT‐mCherry‐transfected PHNs, indicating presence of PDHc in excitatory neurons. In contrast, control autoantibody‐negative human serum showed no colocalization. (B) Representative images of Pat 3 serum incubated on PHNs. Anti‐PDHc Ab‐positive serum showed colocalization with MitoTracker indicating binding to the PDHc within intact mitochondria. Ab‐negative human serum showed no colocalization with the MitoTracker. Scale bars: 50 μm. ctrl, control; DAPI, 4,6‐diamidino‐2‐phenylindole.

To further distinguish PDHc expression on inhibitory and excitatory neurons, PHNs were transduced with markers for inhibitory neurons (rAAV‐mDlx‐Fishell‐GFP) and excitatory neurons (rAAV‐VGLUT2‐mCherry). Sera from PDH Ab‐positive patients bound to both inhibitory and excitatory neurons, with stronger labeling observed in inhibitory interneurons (Figure 3A), consistent with the commercial antibody control (PDH). To confirm the mitochondrial localization of the PDHc and exclude unspecific binding outside of mitochondria, PHNs were labeled with a mitochondrial dye (MitoTracker) and stained with patient sera positive for anti‐PDH Abs (Figure 3B). Strong colocalization between the mitochondrial dye and patient Abs was observed in both the somata and dendrites.

### 3.4 Neuronal uptake of anti‐PDH antibodies in vitro is associated with reduced neuronal viability

**FIGURE 4:**
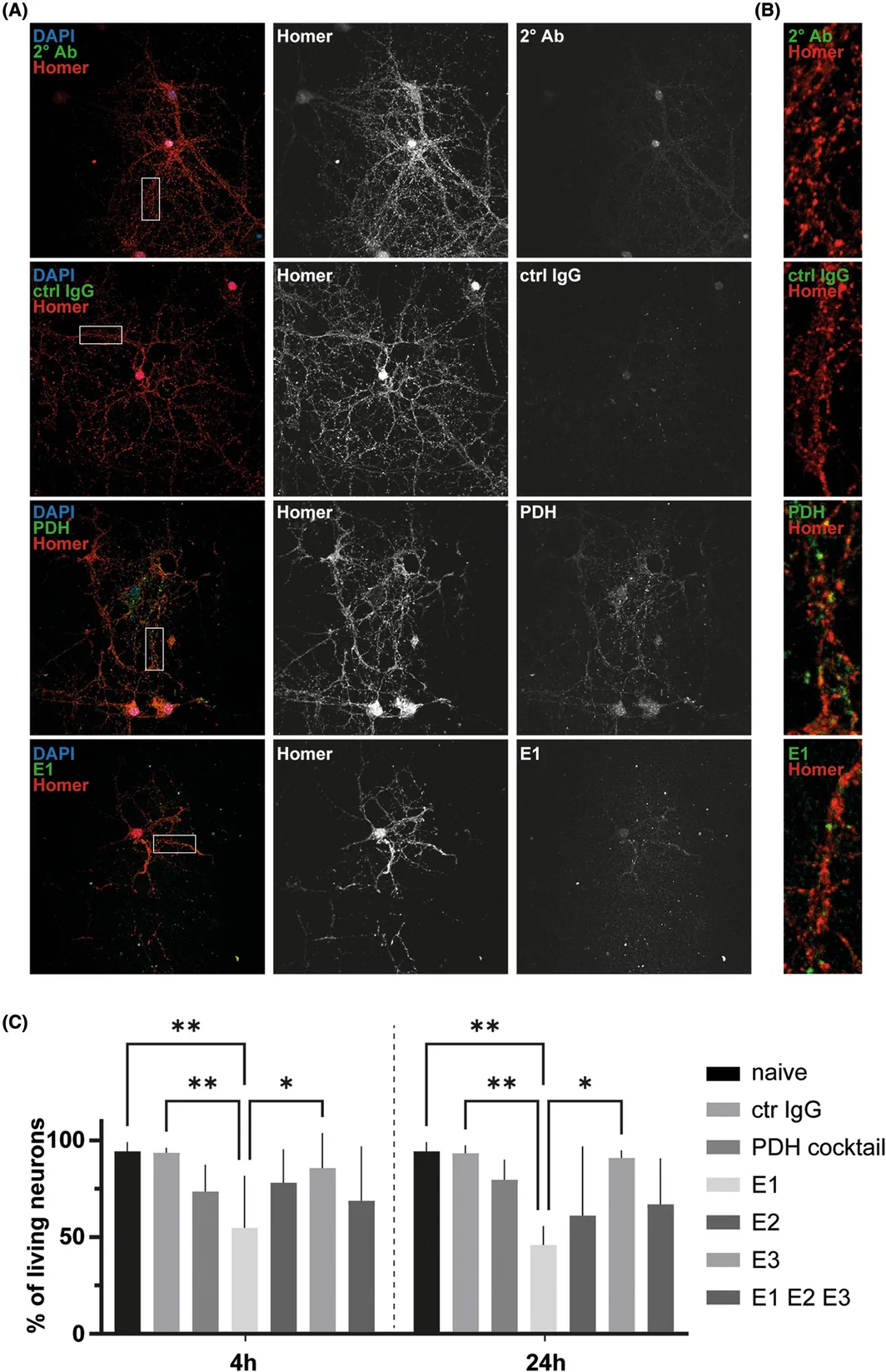
Neuronal uptake of anti‐pyruvate dehydrogenase (PDH) in vitro was accompanied by loss of neuronal processes and reduced neuronal viability. (A) Specific neuronal uptake of anti‐PDH cocktail autoantibodies (Abs) as well as anti‐PDH E1 Ab induced a loss of neuronal processes and (B) neuronal synapses. Antihomer antibody was utilized to visualize neuronal dendrites and synapses. (C) Neuronal viability was reduced after incubation times of both 4 h and 24 h with anti‐PDH E1 Abs (two‐way analysis of variance: treatment, **p = .0005, *F*_6, 28_ = 4.812; incubation time, *p* = .6687, *F*_1, 28_ = .1871; Sidak post hoc test: naïve vs. E1, **p = .0045; control [ctrl] IgG vs. E1, **p = .0057; E1 vs. E3, *p = .0215). DAPI, 4,6‐diamidino‐2‐phenylindole.

For the Abs to reach their target within mitochondria, they must first enter the neuron. To assess neuronal uptake, live PHNs were incubated with commercially available anti‐PDHc Abs and compared to controls. Neuronal uptake of specific anti‐PDH E1 subunit antibodies occurred after 8 h of incubation with minimal uptake as early as after 4 h of incubation, whereas control IgG was not taken up (Figure 4A,B). Furthermore, specific anti‐E2 and anti‐E3 subunit antibodies were not taken up either (data not shown). Following antibody uptake, neurons exhibited reduced arborization. To determine whether uptake of anti‐PDH Ab leads to neuronal death, we measured neuronal viability after 4 h and 24 h of incubation (Figure 4C). Notably, exposure to anti‐E1 subunit Abs significantly reduced neuronal viability, whereas control IgG had no effect.

### 3.5 Increased mtDNA copy number in the peripheral blood of patients with anti‐PDH Abs

**FIGURE 5:**
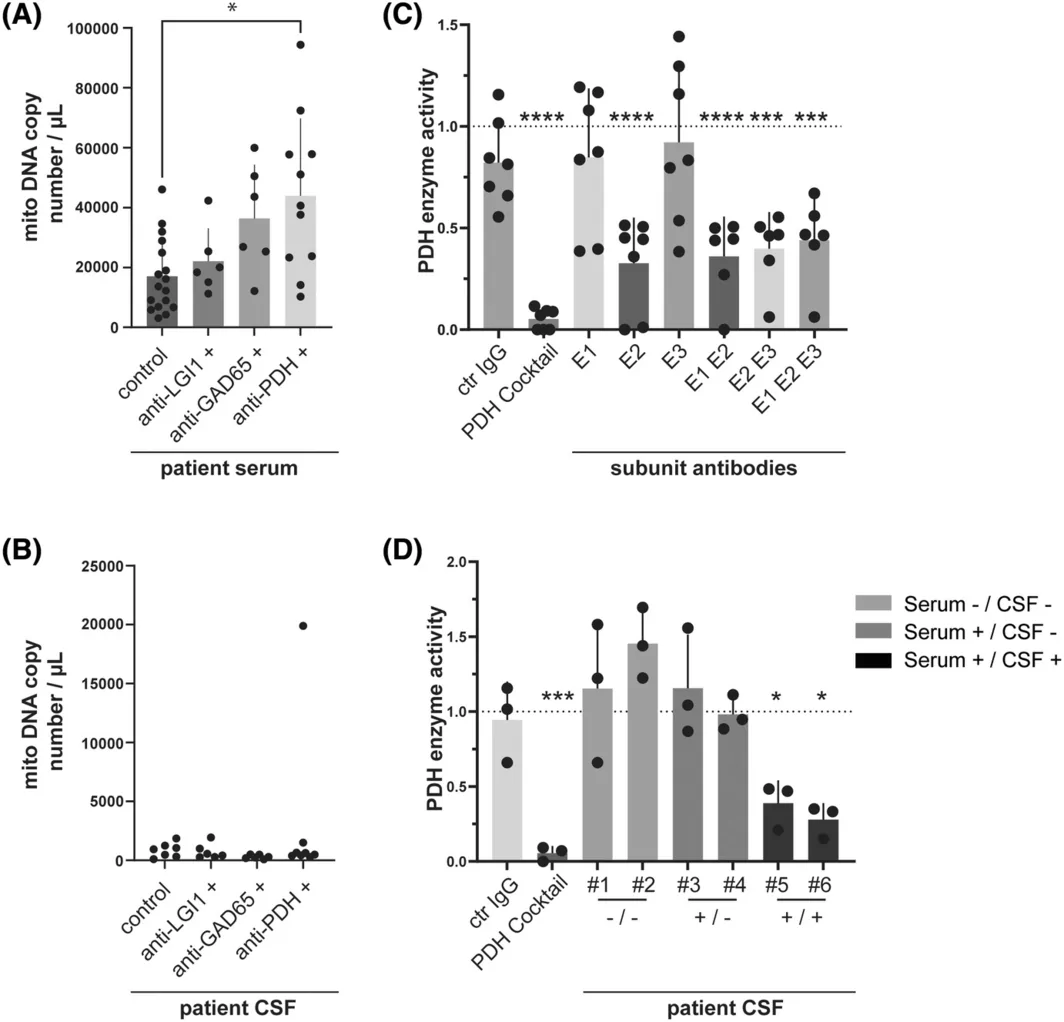
Mitochondrial DNA copy number was increased in anti‐pyruvate dehydrogenase complex (PDHc) autoantibody‐positive serum. Anti‐PDHc autoantibodies in cerebrospinal fluid (CSF) of patients impaired PDHc activity in vitro. (A) Mitochondrial DNA copy number was significantly increased in anti‐PDHc autoantibody‐positive compared to control serum (Kruskal–Wallis test: **p = .0076; post hoc Dunn test: control vs. anti‐PDH+, *p = .0108). (B) The mitochondrial DNA copy number was increased in one of nine CSF samples from patients positive for anti‐PDHc autoantibodies in their serum (Kruskal–Wallis test: *p* = .1817). (C) PDHc enzyme from isolated HEK 293 mitochondria was used for the PDH activity assay. Commercial antibodies against PDHc subunit E2 protein impaired PDH enzyme activity in vitro, indicated by less nicotinamide adenine dinucleotide generated. Anti‐PDHc antibodies as well as anti‐PDH E2 subunit antibodies in combination with other PDH subunit antibodies significantly impaired enzymatic function (one‐way analysis of variance [ANOVA]: ****p < .0001, *F*_8, 51_ = 13.73; Dunnett multiple comparison: PDH cocktail, ****p < .0001; E2, ****p < .0001; E1 vs. E2, ****p < .0001; E2 vs. E3, ***p = .0002; E1 vs. E2 vs. E3, ***p = .0005). (D) Anti‐PDHc autoantibody‐positive CSF significantly impaired enzymatic function, whereas control CSF did not impair PDH enzyme function (one‐way ANOVA: ****p < .0001, *F*_8, 18_ = 11.53; Dunnett multiple comparison: PDH cocktail, ***p = .0009; PDH serum+/CSF+, *p = .0338; PDH serum+/CSF+ (pat #5 and pat #6), *p = .0104). ctr, control; GAD65, glutamic acid decarboxylase 65‐kD isoform; LGI1, leucine‐rich glioma‐inactivated 1; mito, mitochondria.

The copy number of mtDNA D‐loop fragments in body fluids is an indicator of cellular stress and potentially mitochondria‐related cell death.^24^ Because PDH is an enzyme with a strict mitochondrial localization, we quantified the levels of mtDNA D‐loop fragments in the serum and CSF of patients positive for anti‐PDH Abs as well as in control samples. An elevated mean mtDNA fragment copy number was restricted in the serum of patients with anti‐PDH Abs and not present in patients positive for surface (LGI1) or intracellular (GAD65) Abs (Figure 5A). Additionally, the mtDNA copy number was significantly increased in the CSF of one PDH Ab‐positive patient, but there was no significant difference in mtDNA copy number between all anti‐PDH‐positive CSF patients when comparing to the control groups (Figure 5B).

### 3.6 Reduced PDH enzyme activity in vitro after PDH Ab exposure

To directly investigate the effect of anti‐PDH Abs on enzyme activity, isolated mitochondria were incubated with commercially available anti‐PDH Abs or CSF from PDH Ab‐positive patients (Figure 5C,D). It was not feasible to perform the enzymatic assay using patient serum, as the assay relies on detecting a color change from clear to yellow, which would interfere with the natural yellow color of the serum. Both commercially available anti‐PDHc Abs and antibodies targeting the E2 subunit of the PDHc significantly reduced PDH enzyme activity. Furthermore, combining anti‐PDH E2 Abs with antibodies targeting other PDH subunits resulted in a similar reduction in enzyme activity (Figure 5C). To confirm these findings and highlight the specificity of this effect, CSF from PDH antibody‐positive and ‐negative patients was tested. CSF from PDH Ab‐positive patients significantly decreased PDH enzyme activity, whereas control CSF had no effect (Figure 5D).

## 4 DISCUSSION

Mitochondrial dysfunction is implicated in epilepsy, promoting energy deficits, oxidative stress, and impaired neurotransmission.^13^ Our study identifies Abs targeting the PDHc in AIE‐suspicious patients with seizures or psychiatric symptoms. These Abs reduce PDH activity under experimental conditions and are associated with reduced neuronal viability in vitro, supporting a potential link between autoimmunity and metabolic processes. Mutations in PDH cause mitochondrial dysfunction and epilepsy^25^, ^26^, ^27^; we extend this by demonstrating that autoimmune targeting of PDH can interfere with enzymatic activity in vitro. Because systematic genetic screening was only performed in one patient included in this study (with no mutation known for mitochondrial dysfunction) and further testing was guided by clinical indication rather than performed comprehensively, genetic causes of epilepsy cannot be fully excluded, so anti‐PDH Abs should be viewed as an associated immunological finding rather than definitive primary disease cause.

### 4.1 Localization of PDHc

PDH is highly expressed in the mitochondrial matrix of all cells, especially in energy‐demanding brain regions like hippocampus and cerebellum.^28^ Immunohistochemistry confirmed PDH expression in both excitatory and inhibitory neurons, predominantly in dendrites and somata, consistent with its critical role in meeting the metabolic demands of the cellular components and its mitochondrial localization. Patient‐derived anti‐PDH Abs bind these high‐energy regions and disrupt enzymatic activity in vitro, suggesting functional involvement beyond biomarker status.

### 4.2 Mechanistic insights into PDHc disruption

The PDHc plays a central role in energy metabolism, with the E1 subunit initiating pyruvate decarboxylation, the E2 subunit facilitating acetyl group transfer, and the E3 subunit maintaining redox balance.^25^, ^26^, ^29^, ^30^ Disruption of these processes by anti‐PDH Abs is likely to induce mitochondrial dysfunction and potentially contributes to hyperexcitability through reduced inhibitory neurotransmission and impaired metabolic support via cellular respiration resulting in seizures. However, whether anti‐PDH Abs are sufficient to induce seizures and epilepsy will require further clarification in future studies.

### 4.3 Regional and cellular susceptibility to anti‐PDH Abs

Mitochondrial dysfunction appears to disproportionately affect energy‐demanding brain regions such as the hippocampus and cerebellum, both of which are critical for cognitive and motor function.^31^, ^32^ In patients with mesial TLE, the hippocampal subfields, particularly the CA3, are characterized by metabolic abnormalities such as reduced N‐acetyl‐aspartate and elevated lactate levels, which indicate a disturbed energy supply and metabolism.^33^ Similarly, cerebellar Purkinje cells, which rely on γ‐aminobutyric acidergic inhibitory neurons with high mitochondrial content, are particularly susceptible to mitochondrial dysfunction.^8^ Our results are consistent with this regional vulnerability, as patient‐derived anti‐PDH Abs show strong reactivity in the hippocampal pyramidal layer and the cerebellar Purkinje cell layer.

At the cellular level, inhibitory interneurons showed higher PDH expression, which is in line with the higher energy demands of these cells. Importantly, our in vitro studies showed that exposure to anti‐PDH Abs led to significant reductions in neuronal viability and enzymatic activity, consistent with observations that disruptions in PDH function impair oxidative decarboxylation of pyruvate, thereby reducing ATP production critical for neuronal survival.^25^, ^34^ This dual effect—hippocampal vulnerability coupled with cellular susceptibility to Ab binding—highlights the mechanistic link between mitochondrial dysfunction, neuronal injury, and the pathophysiology of epilepsy and other neuropsychiatric disorders in AIE.

### 4.4 Intracellular uptake of anti‐PDH Abs and consequences for neuronal function

Our results demonstrate that anti‐PDH Abs can enter neurons under experimental conditions and are associated with reduced neuronal viability in vitro, supporting their potential functional relevance. AMAs, particularly those targeting PDH, are classically described in primary biliary cirrhosis (PBC), where they are widely regarded as a diagnostic marker without a clearly established direct pathogenic role. In this context, the present findings extend this concept by suggesting that, under defined experimental conditions, such antibodies may exert functional effects on neuronal cells.

In addition, antibody‐mediated inhibition of PDH activity was demonstrated in permeabilized mitochondrial preparations, indicating that AMAs can interfere with enzymatic function when antigen access is experimentally permitted. However, this does not imply that intact IgG readily reaches the mitochondrial matrix in living neurons. Accordingly, direct mitochondrial metabolic impairment in intact neurons remains to be established.

Potential mechanism include interaction with newly synthesized cytoplasmic precursor proteins, binding to mitochondrial‐associated components prior to import, and indirect effects following antibody internalization. However, direct evidence of intracellular trafficking to mitochondria and Fab‐mediated binding to the native target antigen within intact neurons was not assessed.

Neurons with high PDH expression, particularly inhibitory interneurons, may be especially vulnerable due to their high metabolic demand. Their impairment could contribute to disinhibition and neuronal hyperexcitability, a hallmark of epilepsy.^35^ Elevated mtDNA levels further support mitochondrial dysfunction and cell death, highlighting how anti‐PDH Abs may impair neuronal survival and contribute to seizures and excitotoxicity.^36^, ^37^

### 4.5 Abs with intracellularly located target structures: Biomarkers or functional agents?

Abs targeting mitochondrial components are not unique to AIE. AMAs are hallmark biomarkers in PBC and are also detected in a subset of patients with systemic sclerosis. In PBC, AMA titers do not correlate with disease progression or severity,^38^, ^39^, ^40^, ^41^, ^42^, ^43^, ^44^, ^45^ supporting their role as biomarkers rather than direct drivers of pathology.

However, emerging evidence suggests that certain Abs, including anti‐PDH Abs, may exert functional effects under specific conditions. For example, anti‐PDH Abs have been reported in patients with schizophrenia,^46^ although functional data remain limited. Recent studies indicate that intracellular Abs can influence neuronal function (e.g., antidrebrin Abs),^16^ whereas others, such as anti‐GAD65 Abs, remain controversial.^47^

Anti‐PDH Abs pose an additional challenge, as they target a protein located within the double‐membrane‐bound mitochondrial compartment. In the present study, we demonstrate neuronal uptake under experimental conditions and show that anti‐PDH Abs reduce PDH activity and neuronal viability in vitro, supporting their potential functional relevance. These findings contribute to the ongoing discussion of whether certain intracellularly directed Abs may exert effects beyond serving as biomarkers. Similar intracellular activity has been reported for anti‐topoisomerase 1 Abs, which have been shown to enter cells and reach nuclear targets, potentially via raft‐dependent pathways.^48^

The inhibition of PDH inhibition in permeabilized mitochondrial preparations indicates that AMAs can interfere with enzymatic function activity when antigen access is experimentally permitted. However, this does not imply that intact IgG can readily cross mitochondrial membranes in vivo. There is no established physiological mechanism by which a fully folded IgG (~150 kDa) traverses the mitochondrial import machinery, which is highly selective for unfolded precursor proteins. Rather, antibody–antigen interaction are more likely to occur under conditions of cellular stress, apoptosis, or membrane disruption. Importantly, direct evidence of intracellular trafficking to mitochondria and Fab‐mediated binding to the native target antigen within intact neurons was not assessed in the present study.

Intrathecal synthesis of AMAs was not systematically assessed, as antibody‐specific indices were not available. Therefore, a peripheral origin with passive transfer across the blood–brain barrier cannot be excluded. Although their detection in CSF and the observed functional effects support potential relevance in CNS autoimmunity, the present data demonstrate association and functional plausibility rather than definitive evidence of intrathecal antibody production. Future studies incorporating comprehensive CSF immune profiling will be required to clarify their origin.

### 4.6 Implications and future directions

The identification of anti‐PDH Abs suggests a potential immune‐metabolic interaction in a subset of patients with seizures or psychiatric symptoms. Future studies are required to determine their diagnostic and prognostic utility and to assess whether immunomodulatory strategies targeting these Abs may influence clinical outcomes. The observed parallels with other autoimmune diseases such as PBC and systemic sclerosis further support shared underlying mechanisms. Increasing evidence indicates that autoimmune processes contribute to neuronal dysfunction and seizure susceptibility in systemic autoimmune diseases, including systemic lupus erythematosus and other systemic disorders.^49^, ^50^, ^51^

These findings highlight the need for further investigations into these connections to deepen our understanding and improve treatment strategies. A limitation is the use of commercially available antibodies, which may not fully recapitulate the properties of endogenous patient‐derived Abs.

This study raises questions about the broader role of intracellular Abs. Unlike surface‐directed Abs, passive transfer of Abs against intracellular neuronal antigens offers limited mechanistic insight. Restricted access to intracellular targets and the blood–brain barrier prevents these Abs from replicating disease‐relevant effects. Moreover, such models fail to capture the essential cytotoxic T cell‐mediated processes that drive pathology. Therefore, passive transfer is not an appropriate model for AIE linked to intracellular Abs, as these Abs likely act as modulators of disease processes rather than direct effector molecules. Additionally, the mechanisms by which these Abs interfere with their intracellular mitochondrial targets remain unclear and merit further investigation.

## 5 CONCLUSIONS

**FIGURE 6:**
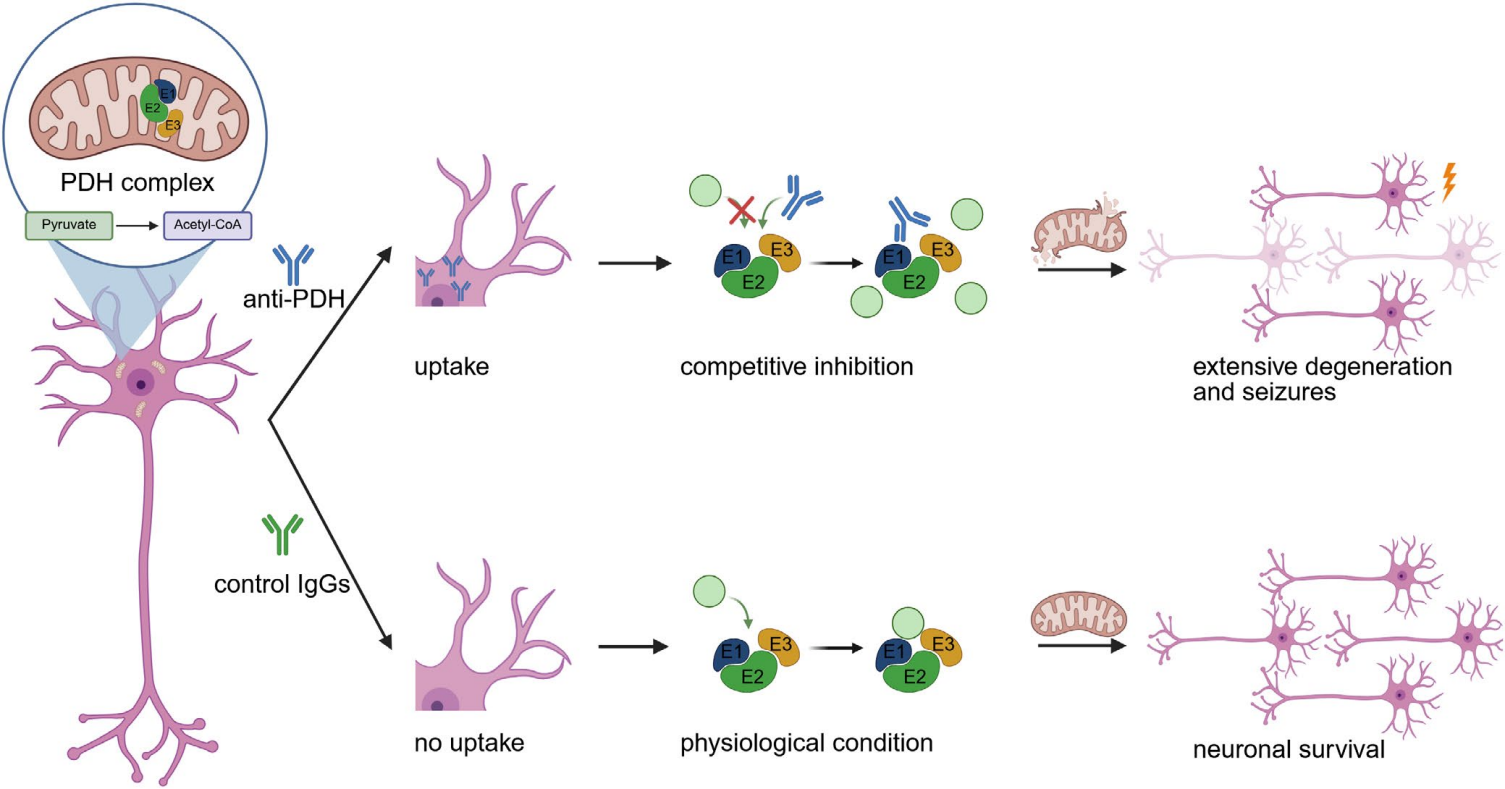
Conceptual model showing functional pathological effects of autoantibodies against pyruvate dehydrogenase (PDH) that inhibit enzyme function and induce neuronal cell death. PDH autoantibodies decrease PDH enzyme activity, thereby inducing extensive neurodegeneration and seizures. Incubation with control IgGs preserves physiological conditions and neuronal survival. Created with BioRender.

Our findings identify anti‐PDH Abs in a subset of patients presenting with seizures and/or psychiatric symptoms in the absence of known neuronal Abs. These Abs bind neuronal structures and reduce PDH enzymatic activity under experimental conditions (Figure 6). Although the in vivo relevance and mechanism of action remain to be clarified, the data support further investigation into potential immunometabolic interactions in this clinical context.

## AUTHOR CONTRIBUTIONS

Annika Breuer, Chiara A. Hummel, Susanne Schoch, Rainer Surges, Albert J. Becker, and Julika Pitsch contributed to the conception and design. Annika Breuer, Chiara A. Hummel, Tobias Baumgartner, Juliane L. Berns, Afaf Milad Said, Isabel Bünger, Gábor Zsurka, Harald Prüss, Wolfram S. Kunz, Albert J. Becker, and Julika Pitsch contributed to the execution or analysis and interpretation of data. Annika Breuer, Chiara A. Hummel, Tobias Baumgartner, Susanne Schoch, Gábor Zsurka, Harald Prüss, Wolfram S. Kunz, Rainer Surges, and Julika Pitsch were involved in drafting and revising the article.

## CONFLICT OF INTEREST STATEMENT

All authors declare no conflict of interest related to this article.

## ETHICS STATEMENT

All procedures were conducted in accordance with the principles of the Declaration of Helsinki. Informed written consent was obtained from every human participant according to the approval of the local ethics committee of the University Hospital Bonn, Bonn, Germany (Nr. 222/16). All animal procedures were planned and performed to minimize pain and suffering, and to reduce the number of animals used in accordance with European, national, and institutional guidelines (guidelines of the European Parliament and of the Council) on the protection of animals used for scientific purposes, European Directive (2010/63/EU), and the ARRIVE guidelines. We confirm that we have read the Journal's position on issues involved in ethical publication and affirm that this report is consistent with those guidelines.

## Supporting information


**Data S1.** Supporting information.

**Table S1.** Antibodies utilized for Western blots, immunohistochemistry, immunocytochemistry, and neuronal uptake assay.

**Table S2.** Three subunits of pyruvate dehydrogenase (PDH) complex are identified by mass spectrometry after immunoprecipitation in three patients with suspected autoimmune encephalitis. Abundance and ratio (abundance sample/abundance controls) of subunits of PDHc identified by mass spectrometry in three index patients are shown. Abundance and ratio of dihydrolipoyllysine‐residue acetyltransferase (DLAT), dihydrolipoamide dehydrogenase (DLD) and PDH E1 component subunit alpha (PDHa1) are markedly increased in Patients 1 and 2 compared to other unspecific identified proteins. Abundance and ratio of DLAT and DLD are markedly increased in Patient 3 in comparison to PDHa1. Ctrl., control; MW, molecular weight; Pat., patient.

**Table S3.** Clinical characteristics of anti‐pyruvate dehydrogenase‐positive patients.

## Data Availability

The data that support the findings of this study are available from the corresponding author upon reasonable request.
