# Supplementary material for: Pyruvate dehydrogenase autoantibodies in autoantibody‐negative patients with seizures are associated with reduced pyruvate dehydrogenase activity
Source: Epilepsia. 2026 May 14;67(8):4078–94. doi: 10.1002/epi.70282 (PMC13525559; doi:10.1002/epi.70282)
Supplement: Supplementary file 1 [file EPI-67-4078-s001.pdf]

Suppl. Table 2: Three subunits of PDH-complex are identified by mass spectrometry after immunoprecipitation in three patients with suspected autoimmune encephalitis.

Abundance and ratio (abundance sample/abundance controls) of subunits of PDH-complex identified by mass spectrometry in three index patients are shown. Abundance and ratio of Dihydrolipoyllysine-residue acetyltransferase (DLAT), Dihydrolipoamide dehydrogenase (DLD) and Pyruvate dehydrogenase E1 component subunit alpha (PDHa1) are markedly increased in Pat #1 and Pat #2 compared to other unspecific identified proteins. Abundance and ratio of DLAT and DLD are markedly increased in Pat #3 in comparison to PDHa1. MW: molecular weight. kDa: kilodaltons. Pat.: patient. Ctrl.: control.

Protein ID	Name	Protein Symbol	PDH subunit	MW [kDa]	Abundance (Pat.)	Abundance (Ctrl.)	Ratio
**Pat.#1**							
Q8BMF4	Dihydrolipoyllysine-residue acetyltransferase	DLAT	E2	67,9	9,2E+09	1,6E+07	5,6E+02
Q8BKZ9	Dihydrolipoamide dehydrogenase	DLD	E3	54	4,5E+07	7,8E+05	5,8E+01
P35486	Pyruvate dehydrogenase E1 component subunit alpha	PDHa1	E1α	43,2	3,3E+10	6,7E+06	4,9E+03
**Pat.#2**							
Q8BMF4	Dihydrolipoyllysine-residue acetyltransferase	DLAT	E2	67,9	7,1E+09	3,7E+07	1,9E+02
Q8BKZ9	Dihydrolipoamide dehydrogenase	DLD	E3	54	8,6E+07	7,8E+05	1,1E+02
P35486	Pyruvate dehydrogenase E1 component subunit alpha	PDHa1	E1α	43,2	4,6E+10	6,7E+06	6,8E+03
**Pat.#3**							
Q8BMF4	Dihydrolipoyllysine-residue acetyltransferase	DLAT	E2	67,9	4,4E+10	1,6E+07	2,7E+03
Q8BKZ9	Dihydrolipoamide dehydrogenase	DLD	E3	54	9,4E+07	1,9E+05	4,8E+02
P35486	Pyruvate dehydrogenase E1 component subunit alpha	PDHa1	E1α	43,2	1,7E+07	8,4E+06	2,0E+00
