# Supplementary material for: Pyruvate dehydrogenase autoantibodies in autoantibody‐negative patients with seizures are associated with reduced pyruvate dehydrogenase activity
Source: Epilepsia. 2026 May 14;67(8):4078–94. doi: 10.1002/epi.70282 (PMC13525559; doi:10.1002/epi.70282)
Supplement: Supplementary file 2 [file EPI-67-4078-s003.pdf]

Suppl. Table 3: Clinical characteristics of anti-PD-positive patients.

**Demographics**	**anti-PDH-positive Patients**
Number of patients (n)	12
Female, n (%)	6 (50)
Age at onset, years (median, range)	52 (13-79)
Age at sampling, years (median, range)	55 (18-79)
**PDH subunits positivity**	n (%)
E1α/ E2/ E3	3 (25)
E1α/ E2	1 (8)
E2/ E3	4 (34)
E2	3 (25)
E3	1 (8)
**Serology / CSF diagnostics**	yes / no: n (%)
CSF PDH positive	2 / 7 (22 / 78)
CSF pathology	5[A] / 5 (50 / 50)
ANA positive	4 / 6 (40 / 60)
Hep2 positive	4 / 8 (33 / 67)
**cMRI**	yes / no: n (%)
cMRI pathology	9 / 3 (75 / 25)
Temporomesial FLAIR/T2 hyperintensities, n	3
hippocampal sclerosis, n	1
cerebral small vessel disease, n	3
other[B], n	2
**Diagnosis**	yes / no: n (%)
temporal lobe epilepsy (TLE), n (%)	8[D] (53)
paroxysmal episodes of unknown etiology, n (%)	1 (7)
Status epilepticus, n (%)	2 (13)
psychiatric disorder[C], n (%)	5[D] (33)
overlap (TLE + psych. disorder)[D]	4 (27)
tumor in medical history, n (%)	2 (13)
comorbid autoimmune disorder[E], n (%)	2 (13)
**Symptoms**	yes / no: n
seizures	10 / 2
epilepsy	9 / 2
psychiatric symptoms	5 / 7
non-epileptic episodes	2 / 10
**Seizure outcome**	yes / no: n
IT	0 / 12
ASM	9 / 3
response rate ≥ 50 %	5
pharmacoresistant	2

A Weak BBB disruption (n=4), pleocytosis (n=1), intrathecal IgG synthesis (n=1)
B Posthaemorrhagic lesion frontal right (n=1), Nodular cortical heterotopia (n=1)
C depression (n=3), bipolar disorder (n=1), post-traumatic stress disorder (n=1)
D patients with TLE had additionally depression (=3) or post-traumatic stress disorder (n=1) as psychiatric disorder
E psoriasis (n=1), Primary biliary cholangitis (PBC; n=1)
IT = immunotherapy, ASM = Anti-seizure medication
