# Supplementary material for: Pyruvate dehydrogenase autoantibodies in autoantibody‐negative patients with seizures are associated with reduced pyruvate dehydrogenase activity
Source: Epilepsia. 2026 May 14;67(8):4078–94. doi: 10.1002/epi.70282 (PMC13525559; doi:10.1002/epi.70282)
Supplement: Supplementary file 3 [file EPI-67-4078-s004.pdf]

Suppl. Table 1: Antibodies utilized for western blots, immunohistochemistry, immunocytochemistry and neuronal uptake assay.

Antibody	Company	Cat. Number	Host species	Usage	Dilution
anti-PDH-complex	Abcam	ab110416	Mouse	1° WB validation	1:1000
anti-human IRDye 800 CW	LI-COR Biosciences	926-32232	Goat	2° WB validation	1:25000
anti-mouse IRDye 800 CW	LI-COR Biosciences	926-68070	Goat	2° WB validation	1:25000

Antibody	Company	Cat. Number	Host species	Usage	Dilution
anti-PDH-complex	Abcam	ab110416	Mouse	1° IHCs	1:200
Alexa Fluor 488 anti-mouse	Life Technologies	A-11001	Goat	2° IHCs	1:200
Alexa Fluor 568 anti-human	Life Technologies	A-21090	Goat	2° IHCs	1:200

Antibody	Company	Cat. Number	Host species	Usage	Dilution
anti-PDH-complex	Abcam	ab110416	Mouse	1° ICC	1:1000
anti-PDH E2	Abcam	ab110332	Mouse	1° ICC	1:500
anti-PDH ED	Abcam	ab172317	Rabbit	1° ICC	1:100
anti-PDH E1a	Sigma Aldrich	AP1064	Rabbit	1° ICC	1:500
anti-PDH E3	Abcam	ab186827	Rabbit	1° ICC	1:500
Alexa Fluor 488 anti-mouse	Life Technologies	A-11001	Goat	2° ICC	1:1000
Alexa Fluor 647 anti-mouse	Life Technologies	A-21236	Goat	2° ICC	1:1000
Alexa Fluor 568 anti-rabbit	Life Technologies	A-11011	Goat	2° ICC	1:1000
Alexa Fluor 568 anti-human	Life Technologies	A-21090	Goat	2° ICC	1:1000

Antibody	Company	Cat. Number	Host species	Usage	Dilution
Anti-Homer1	Synaptic Systems	160004	Guinea pig	1° ICC	1:1000
anti-PDH-complex	Abcam	ab110416	Mouse	Uptake assay	6 µg
anti-PDH E1	Novus Biologicals	NB110-93479	Rabbit	Uptake assay	6 µg
anti-PDH E2	Abcam	ab110332	Mouse	Uptake assay	6 µg
anti-PDH E3	Abcam	ab186827	Rabbit	Uptake assay	6 µg
intratect	Biotest	PZN 03433745	Human	Uptake assay	6 µg
Alexa Fluor 488 anti-mouse	Life Technologies	A-11001	Goat	2° ICC	1:1000
Alexa Fluor 488 anti-rabbit	Life Technologies	A-11008	Goat	2° ICC	1:1000
Alexa Fluor 568 anti-guinea pig	Life Technologies	A-11073	Goat	2° ICC	1:1000
Alexa Fluor 488 anti-human	Life Technologies	A-11013	Goat	2° ICC	1:1000
