# Supplementary material for: Pyruvate dehydrogenase autoantibodies in autoantibody‐negative patients with seizures are associated with reduced pyruvate dehydrogenase activity
Source: Epilepsia. 2026 May 14;67(8):4078–94. doi: 10.1002/epi.70282 (PMC13525559; doi:10.1002/epi.70282)
Supplement: Supplementary file 4 [file EPI-67-4078-s002.docx]

**Pyruvate dehydrogenase autoantibodies in autoantibody-negative patients with seizures are associated with reduced pyruvate dehydrogenase activity**

Annika Breuer^1#^, Chiara A. Hummel^1#^, Tobias Baumgartner^1^, Juliane L. Berns^1^, Afaf Milad Said^2^, Isabel Bünger^4^, Susanne Schoch^1,3^, Gábor Zsurka^1,2^, Harald Prüss^5,6^, Wolfram S. Kunz^1,2^, Rainer Surges^1^, Albert J. Becker^3#^, Julika Pitsch^1#*^

^1^Department of Epileptology, University Hospital Bonn, Bonn, Germany,

^2^Inst. of Experimental Epileptology and Cognition Research, University Hospital Bonn, Bonn, Germany,

^3^Institute of Cellular Neurosciences II, University Hospital Bonn, Bonn, Germany,

^4^Department of Pediatric Neurology, Charité Universitätsmedizin Berlin, Berlin, Germany,

^5^Department of Neurology and Experimental Neurology, Charité Universitätsmedizin Berlin, Berlin, Germany,

^6^German Center for Neurodegenerative Diseases (DZNE) Berlin, Berlin, Germany

**Supplementary methods**

**Screening for antineuronal autoantibodies**. Using commercial diagnostic kits, all analyzed serum and CSF samples were tested for neuroABs known to be associated with LE or other neuroimmunological syndromes. Specifically, we tested for “onconeuronal” autoABs against amphiphysin, CV2, PNMA2 (Ma2/Ta; paraneoplastic antigen Ma2), Ri, Yo, Hu, Recoverin, SOX1, Titin, Zic4 (zinc finger protein Zic4), GAD65 (glutamic acid decarboxylase 65) and Tr (DNER) using semi-quantitative immunoblots according to the manufacturer's guidelines (EUROLINE PNS 12, Euroimmun, DL 1111-1601-7 G), coated with recombinant antigen or antigen fragments with serum 1: 100 and CSF 1:4 (sensitivity: 89-100%; specificity: 99%, according to the manufacturer's instructions). In addition, indirect immunofluorescence was performed on HEK293T cells (Human Embryonic Kidney 293), which overexpress the individual antigens on the cell surface (IIFT: 'Autoimmune Encephalitis Mosaic1', Euroimmun, FA 1120-1005-1; GAD65-IIFT, Euroimmun, FA 1022-1005-50) for anti-NMDAR, anti-CASPR2, anti-LGI1, anti-GABA_A_R, anti-GABA_B_R, anti-AMPAR and anti-GAD65 AutoABs (dilution: Serum 1:10; CSF undiluted) according to the manufacturer's protocol (sensitivity: 82-100%; specificity: 98.3-100%, according to the manufacturer's instructions).
